# Assessing the impact of socioeconomic status on incidental lung nodules at an urban safety net hospital

**DOI:** 10.1186/s12890-023-02726-8

**Published:** 2023-11-23

**Authors:** Mateus Fernandes, Cristian Milla, Ahmed Gubran, Sandra Barrazueta, Brian Altonen, Anthony DiVittis, Woodhull Resident Research Team, Prama Rashmi, Olva Bess, Haris Asif, Ifediba Nwachukwu, Opeyemi Aroyewun, Alberto Martinez, Stephen Kuperberg

**Affiliations:** 1 Pulmonary and Critical Care Medicine, Lenox Hill Hospital, Northwell Health, New York, USA; 2 Department of Medicine, NYC Health + Hospitals/Woodhull, New York City Health and Hospitals, 760 Broadway, Brooklyn, NY 11206 USA; 3 Division of Nephrology, SUNY Downstate/Health Sciences Center at Brooklyn, NY Brooklyn, USA; 4 Research and Administration, New York City Health and Hospitals, NY New York, USA; 5 Pulmonary and Critical Care Medicine, New York University Grossman School of Medicine, NY New York, USA

**Keywords:** Lung nodule, Lung cancer, Inequity, Disparity, Socioeconomic, Disadvantage, Deprivation

## Abstract

**Introduction:**

Lower socioeconomic status has been identified as an emerging risk factor for health disparities, including lung cancer outcomes. Most research investigating these outcomes includes patients from formal lung cancer screening programs. There is a paucity of studies assessing the relationship between socioeconomic status and incidental lung nodules. This study aimed to investigate the association between socioeconomic status and the size of incidental lung nodules on initial presentation at an urban safety net hospital, which did not have a formal lung cancer screening program or incidental lung nodule program.

**Methods:**

A retrospective chart review was conducted on patients with incidental lung nodules on CT chest imaging who were referred from primary care to a pulmonology clinic at a safety net hospital. Patients with incomplete nodule characteristics information were excluded. Data on demographics, comorbidities, smoking history, insurance type, immigration status, and geographical factors were collected. Less commonly studied determinants such as crime index, cost of living, and air quality index were also assessed. Logistic regression analysis was performed to assess relationships between nodule size and socioeconomic determinants.

**Results:**

Out of 3,490 patients with chest CT scans, 268 patients with ILNs were included in the study. 84.7% of patients represented racial or ethnic minorities, and most patients (67.8%) had federal insurance. Patients with non-commercial insurance were more likely to have larger, inherently higher-risk nodules (> 8 mm) compared to those with commercial insurance (OR 2.18, *p* 0.01). Patients from areas with higher unemployment rates were also less likely (OR 0.75, *p* 0.04) to have smaller nodules (< 6 mm). Patients representing racial or ethnic minorities were also more likely to have nodules > 8 mm (OR 1.6, p 0.24), and less likely to have nodules < 6 mm (OR 0.6, *p* 0.32), however, these relationships were not statistically significant.

**Conclusion:**

This study found that lower socioeconomic status, indicated by having non-commercial insurance, was associated with larger incidental lung nodule size on initial presentation. While it is established that socioeconomic status is associated with disparities in lung cancer screening, these findings suggest that inequalities may also be present in those with incidental lung nodules. Further research is needed to understand the underlying mechanisms and develop interventions to address these disparities in incidental lung nodule evaluation and improve outcomes.

## Introduction

Despite global collaborative efforts in prevention and management, lung cancer continues to be the leading cause of cancer-related deaths worldwide [1]. On the basis of strong evidence [2–7], a key intervention now widely employed to attenuate the impact of lung cancer is lung cancer screening (LCS), which serves as a public health measure aiming to identify high-risk individuals at an early stage and thus confer favorable potential for surgical intervention and survival [2, 4]. Indeed, multiple randomized trials have confirmed the value of lung cancer screening in reducing lung cancer mortality in high-risk individuals via early detection [5–7]. However, an increasing number of lung nodules are detected incidentally. As computed tomography (CT) chest imaging has become more common, there has been a parallel increase in the detection of incidental lung nodules (ILNs) [8]. ILNs are now a common finding with a prevalence of 10% to 30% on chest CT scans [9]. Individuals with ILNs have different characteristics and smoking behavior compared to those eligible for LCS, with less than half meeting the updated USPSTF screening criteria [10]. Therefore, LCS and ILN surveillance programs seem to reach different but complementary at-risk populations [9, 10].

In both LCS and ILN surveillance, guideline concordant evaluation and follow-up rates are low [11]. Unfortunately, significant disparities have been uncovered based on race, income, and geographic location in LCS programs. However, there is a paucity of studies evaluating how social determinants of health are related to ILNs [11]. Studies have shown that individuals with ILNs are more likely to be African American, nonsmokers or have stopped smoking [9, 12]. Other authors have demonstrated that individuals from ILN programs who were diagnosed with cancer were more likely to be uninsured or have federal insurance [10], providing further evidence of SES inequalities. For this reason, diverse efforts are being made to prioritize these socioeconomically driven challenges and overcome barriers to optimal lung cancer evaluation [11]. The objective of this study was to assess the impact of poverty and SES on incidental lung nodule size in an urban, socioeconomically deprived population of high-risk individuals.

## Methods

### Study design

The aim of the study was to determine whether lower socioeconomic status (SES) was associated with larger nodules on initial presentation. We assessed the relationships between determinants of SES and incidental nodule size. We hypothesized that individuals with markers of lower SES were more likely to present with larger, higher risk incidental nodules. NYC Health + Hospitals/Woodhull, a community hospital within the New York City Health and Hospitals (NYCHHC) network, was the primary study location. NYCHHC is the largest municipal provider of safety-net care in the United States, serving more than one million city residents who either utilize federal insurance, are uninsured or undocumented [13]. In addition, Woodhull is situated in a designated healthcare professional shortage area in Brooklyn, NY, where a significant proportion of the population is uninsured or underinsured. The center had no formal LCS or ILN surveillance program during the study period, and individuals were not enrolled in external LCS or ILN programs.

We performed a retrospective chart review of individuals ≥ 18 years of age who were referred from primary care services to the pulmonology clinic from February 2019 to February 2022. Of these individuals, we included all referrals with newly discovered, incidental lung nodules seen on CT chest, since a formal lung cancer screening program was not in place at our facility during this timeframe. Individuals with nodules identified on chest radiographs only, and those with incomplete description or missing nodule measurements were excluded. For example, if the nodules were described as “small”, or no size was reported, then the nodule as excluded. A lung nodule was classified as an incidental nodule if there was no record of a lung mass or nodule in the patient's medical history, including previous imaging reports and clinical notes. Pulmonary nodules were classified as any lesion that measured less than 30 mm located in the pulmonary parenchyma. The size of nodules was recorded in millimeters of greatest diameter, measured linearly on two-dimensional imaging. When multiple nodules were present, the largest nodule diameter was recorded for analysis. The nodule size recorded for analysis was determined by the radiologist report of the CT chest scan.

Approval by the New York University School of Medicine Institutional Review Board was obtained prior to the initiation of the study.

Nodule sizes were grouped into three tiers of risk per Fleischner Society Guidelines for evaluation of the solitary pulmonary nodule [14]. Recommendations for follow-up were based on nodule size being less than 6 mm, between 6 and 8 mm, and greater than 8 mm [14]. We allocated nodules to each of these categories for analysis.

### Data collection

Data were collected using electronic medical record review. The data were initially collected by members of the research team and then reviewed for accuracy by the primary author. Individual patient charts were reviewed to obtain relevant information, including demographics (age, gender, ethnicity, race, zip code), comorbidities, smoking history, and determinants of socioeconomic status. Insurance type and immigration status were obtained from charts. Determinants of SES derived from the individual patient chart were classified as individual markers of SES. The United States 2020 Census data were used to obtain median income, educational attainment, percentage of those living below the federal poverty line, place of birth and unemployment rate according to zip code. A public data repository, City-data [15], was used to obtain data on the air quality index, cost of living index, and violent crime index, according to patient zip code. Determinants of SES based on zip code, using census data and the data-repository, were classified as geographic markers of SES.

### Statistical analysis

Statistical analysis was performed using JASP (version 0.16.4), which is a computer-based statistics program. Descriptive statistics on frequencies and medians were obtained to describe our study sample and nodule characteristics. Logistic regression was used to obtain adjusted odds ratios assessing the relationship between nodule size (dependent variable) and SES determinants (covariates) for individual and geographical markers of SES. A logistic regression model was used for analysis of each of the three nodule size categories. For each model, patients belonging to the same nodule category were coded as ‘yes’, while other patients were coded as ‘no’. For example, for the < 6 mm model, patients with ILNs less than 6 mm were coded as ‘yes’ while patients from the other two categories (6 mm to 8 mm, and greater than 8 mm) were coded as ‘no’. Independent variables consisted of race/ethnicity, insurance type, immigration status, and geographical factors including median income, poverty level, cost of living index, air quality index, violent crime index, educational attainment, and unemployment rate. Insurance type was defined by one composite group labeled ‘noncommercial insurance’, which included individuals without commercial insurance and uninsured individuals. Race was defined by one composite group labeled ‘nonwhite race’, which included all individuals of races other than white. Odds ratios derived from the logistic regression models were adjusted for covariates including age in years, sex (male as reference), smoking status (current or former smoker), and history of COPD. A *p* value of < 0.05 was determined to be statistically significant, and the effect size was determined by odds ratios.

### Data reporting

The study was designed and reported according to the “Strengthening The Reporting of Observational Studies in Epidemiology” (STROBE) guidelines. A checklist indicating STROBE components and page number is provided in the appendix. Demographics and patient characteristics were reported in Table 1. Results from the logistic regression models were reported in two separate tables (Tables 3 and 4), grouped by relevant variables (individual and geographical determinants).

**Table 1:** Demographics, comorbidities, and nodule characteristics of individuals with incidental nodules

**Demographics**	
**Age** (years)	
Median 25^th^ to 75^th^ percentile	65 58 to 74
**Sex**	
Male	145 (54.1%)
Female	123 (45.9%)
**Race/Ethnicity**	
African American	71 (26.5%)
Asian	6 (2.2%)
Nonwhite Hispanic	150 (56.0%)
White	41 (15.3%)
**Comorbidities**	
Hypertension	180 (67.2%)
Diabetes	86 (32.1%)
Asthma	73 (27.2%)
COPD	93 (34.7%)
Current/former smoker^a^	200 (74.5%)
History of COVID-19	54 (20.1%)
**Nodules/Mass Characteristics**	
Nodule Size (mm) Median 25 th to 75 th percentile	9 5 to 16
Nodule < 6 mm	
Nodule 6–8 mm	56 (20.9%)
Nodule > 8 mm	109 (40.7%)
Mass (> 30 mm)	29 (10.8%)

^a^Current/former smoker, 2 individuals missing data

## Results

**Fig. 1:**
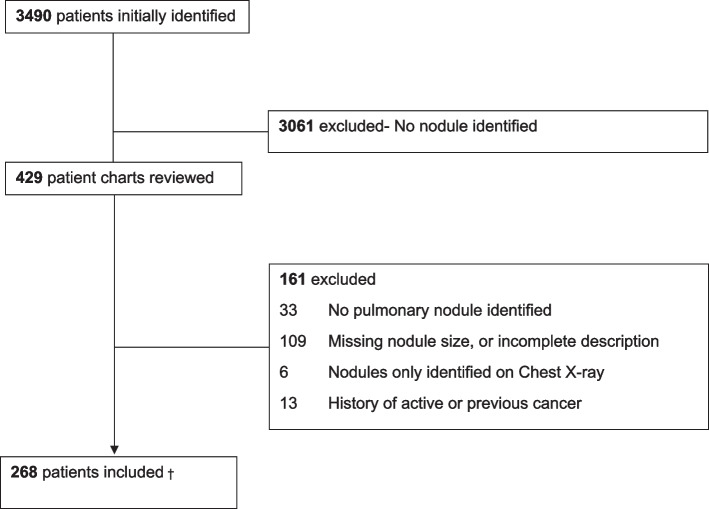
Flow chart of the study, †This includes 239 individuals with nodules and 29 individuals with lung masses

There were 3490 individuals with chest CT scans between February 2019 and February 2022 who were referred to the pulmonology clinic. After exclusion criteria were applied, 3222 individuals were excluded due to various criteria, most common being the absence of nodules or missing nodule data. A total of 268 individuals were included in the final study (Fig. 1).

Individuals were mostly within the age group 58–74 years with a median of 65 years. Most individuals were from a racial or ethnic minority group (84%), with the largest group being nonwhite Hispanic individuals (56%), followed by African American, White, and Asian individuals. Comorbidities were prevalent, most commonly hypertension followed by COPD. There was a high prevalence of tobacco use at 74.5%. There was a high prevalence of clinically significant lesions (defined as nodules with size 6 mm or greater) at 72.4%, most of which were 8 mm or larger.

Table 2 highlights geographical and individual socioeconomic determinants. Most individuals were either using federal insurance or uninsured (73%). Geographical factors were also measured and analyzed for associations with nodule size.

**Table 2:** Socioeconomic determinants according to insurance type and geographical area

Socioeconomic Determinants	
**Insurance Type (%)**	
Commercial	72 (26.9%)
Medicare	19 (7.1%)
Medicaid	135 (50.3%)
Medicare/Medicaid	10 (3.7%)
NYC Care ^a^	18 (6.7%)
Uninsured	14 (5.2%)
**Geographical Factors** (Median)	
Violent Crime Index	198
Cost of living Index	123
Air Quality Index	38.15
Birthplace outside US (%)	23.9
Educational Attainment Less than High School/Equivalent (%)	17.3
Living Below Federal Poverty Level (%)	25.7
Unemployment Rate (%)	8.12

^a^NYC care is a unique type of federal insurance offered exclusively to undocumented individuals in New York City

Table 3 shows associations of nodule size with various factors using logistic regression analysis, with covariates of age, sex, tobacco use and comorbidities.

**Table 3:** Impact of individual socioeconomic determinants and comorbidities on incidental lung nodule size

	**Nodule < 6 mm**			**Nodule 6–8 mm**			**Nodule > 8 mm**		
**Variable**	Odds Ratio	95% Confidence Interval	*p* value	Odds Ratio	95% Confidence Interval	*p* value	Odds Ratio	95% Confidence Interval	*p* value
**Demographics**									
Age	0.985	0.918, 1.014	0.073	0.987	0.92, 1.023	0.278	1.024	1.007, 1.107	0.027
Male	0.569	0.076, 1.099	0.056	1.965	1.089, 20.65	0.038	0.965	0.266, 3.192	0.898
Nonwhite race	0.668	0.062, 2.523	0.327	0.899	0.099, 6.209	0.817	1.627	0.469, 20.05	0.242
**SES Determinants**									
Undocumented	1.185	0.843, 20.61	0.771	0.795	0.028, 12.62	0.735	1.026	0.095, 11.89	0.962
Noncommercial Insurance	0.437	0.035, 0.637	0.01	1.03	0.208. 5.495	0.935	2.181	1.544, 2.818	0.016
**Comorbidities**									
Tobacco Use	0.971	0.196, 4.446	0.932	1.129	0.221, 7.907	0.76	0.95	0.204, 3.873	0.875
Diabetes	1.113	0.274, 5.998	0.754	0.962	0.159, 5.248	0.92	0.94	0.21, 3.59	0.845
Hypertension	1.32	0.365, 9.863	0.447	0.667	0.066, 2.344	0.306	1.051	0.244, 5.14	0.884
Asthma	0.952	0.208, 3.828	0.878	1.3	0.372, 9.016	0.457	0.867	0.185, 2.805	0.635
COPD	1.107	0.288, 5.534	0.756	0.902	0.153, 4.055	0.776	0.981	0.246, 3.724	0.95
HIV	1.474	0.179, 33.34	0.503	0.546	0.007, 9.099	0.448	1.004	0.078, 13.12	0.994

Increased age was associated with nodules > 8 mm (OR 1.024, *p* 0.042); however, the effect size was negligible. Male individuals were less likely to have nodules < 6 mm (OR 0.56, *p* 0.056) and more likely to have nodules between 6–8 mm (OR 2.039, *p* 0.033). Individuals with noncommercial insurance were less likely to have nodules less than 6 mm (OR 0.437, *p* 0.01). Having noncommercial insurance was also associated with nodules > 8 mm (OR 2.181 *p* 0.016).

Table 4 shows the relationships between geographical SES determinants and nodule size using logistic regression adjusted for age, sex (male reference), history of COPD, and tobacco use. Individuals from areas with a higher rate of non-US birthplaces were slightly less likely to have nodules < 6 mm (OR 0.896, *p* 0.015) and more likely to have nodules > 8 mm (OR 1.078, *p* 0.051). Individuals from areas with lower educational attainment were more likely to have nodules < 6 mm (OR 1.243, *p* 0.008) and less likely to have nodules between 6–8 mm (OR 0.795, *p* 0.027). A lower unemployment rate was associated with smaller nodules < 6 mm (OR 0.754, *p* 0.045). Individuals from areas with lower income were less likely to have nodules 6–8 mm (OR 0.96, *p* value 0.02).

**Table 4:** Impact of socioeconomic determinants at the geographic level on incidental lung nodule size

	**Nodule < 6 mm**			**Nodule 6–8 mm**			**Nodule > 8 mm**		
	Odds Ratio	95% Confidence Interval	*p* value	Odds Ratio	95% Confidence Interval	*p* value	Odds Ratio	95% Confidence Interval	*p* value
Birthplace outside US ^a^	0.896	0.632, 0.953	0.015	1.014	0.826, 1.291	0.776	1.078	0.998, 1.413	0.051
Violent Crime Index	1.016	0.966, 1.112	0.314	0.997	0.923, 1.072	0.877	0.992	0.891, 1.084	0.723
Cost of Living Index	0.933	0.724, 1.005	0.056	1.084	1.002, 1.445	0.048	0.993	0.849, 1.14	0.823
Air Quality Index	0.466	0.006, 5.105	0.309	1.576	0.085, 95.94	0.56	1.848	0.17, 99.31	0.384
Education ^b^	1.243	1.143, 2.382	0.008	0.795	0.37, 0.942	0.027	0.949	0.638, 1.227	0.467
Poverty Level ^c^	0.973	0.767, 1.151	0.543	0.942	0.7, 1.084	0.213	1.084	0.984, 1.469	0.071
Unemployment Rate	0.754	0.276, 0.986	0.045	1.143	0.746, 2.483	0.314	1.114	0.755, 2.178	0.357
Median Income	1.007	0.951, 1.089	0.633	0.96	0.838, 0.991	0.028	1.018	0.977, 1.112	0.208

^a^Birthplace outside the US is defined by the percentage of individuals with a place of birth listed as foreign according to the United States 2020 Census data

^b^Education was defined by the percentage of individuals with less than a high school level or equivalent education

^c^Poverty level was defined by the percentage of individuals living below the federal poverty level. Odds ratios were adjusted for covariates including age, smoking status, history of COPD and sex (with male as the reference)

## Discussion

Social determinants of health have a significant impact on diverse health outcomes, including malignancy. There is emerging awareness of socioeconomic status and socioeconomic deprivation as a primary risk factor for both incidence and adverse outcomes in lung cancer [16–18]. SES is comprised of a broad, heterogeneous yet crucial set of factors used to measure an individual’s social and economic standing, built on parameters such as income, education level, insurance carrier, and geographic location [19, 20]. While there is a gap in literature on studies evaluating the impact of SES on ILN outcomes, robust evidence suggests that individuals with lower SES present with more advanced-stage lung cancer at diagnosis, attenuated response to chemotherapy and obtain less favorable prognosis after diagnosis [16, 18]. There is also a lack of trials evaluating outcomes of ILN programs. However, large trials that investigated lung cancer screening, e.g., the National Lung Screening Trial (NLST) and the Dutch-Belgian lung cancer screening [Nederlands–Leuvens Longkanker Screenings Onderzoek (NELSON)] trial, demonstrated a significant mortality reduction of 20–26% [5–7]. Notably, sociodemographic inequalities were present in these trials, and thus there are limitations in generalizing the results to diverse populations with respect to ethnicity, race and income level. Participants in NSLT were predominantly of white race (91%) and had higher educational status (32% with college degrees) and income compared to the general population matched to age and smoking criteria [21]. Therefore, it is uncertain whether the benefits seen in these trials are applicable to those with lower SES.

While numerous studies have evaluated socioeconomic disparities among individuals enrolled in or eligible for LCS programs, there is a paucity of literature on the clinical impact of SES on incidental lung nodule evaluation and follow up [11]. Lower SES is associated with lower LCS adherence, utilization, and guideline concordant care, yet to date, there is minimal data on the impact of SES on ILNs. In the DELUGE study, which prospectively evaluated both LCS and ILN surveillance, individuals from the lung nodule surveillance program were more likely to be diagnosed with lung cancer [10]. Other studies have highlighted the advantages of combined LCS and ILN programs [9, 10, 12, 22] to capture higher-risk groups, which leads to improved early lung cancer diagnosis and guideline concordant care. However, SES factors in these studies were not consistently reported, and these studies included mostly individuals of White race, which made it challenging to evaluate the effect of the programs when accounting for SES, race, and ethnic inequalities [23]. In our study, we aimed to elucidate the influence of SES on incidental lung nodule characteristics. We performed a retrospective analysis of all individuals ≥ 18 years of age with incidental lung nodules on CT chest who were referred to the Pulmonary clinic at an urban, safety net hospital in an underserved area of Brooklyn, New York over a 3-year period. Such hospitals are an essential safety net serving a socioeconomically diverse population, wherein individuals are typically less likely to be included in incidental lung nodule programs or participate in clinical trials [16]. Specifically, we aimed to evaluate the association between incidental nodule size and SES status using common determinants such as education, insurance type and income, as well as less commonly used indicators such as air quality index, cost of living index and immigration status.

### Socioeconomic status and nodule size

The impact of SES on nodule size was assessed with logistic regression analysis using individual and geographical determinants. Categorization of SES by geographic area has gained traction in recent years with the development of indices such as the area-based deprivation index (ADI) [20]. Geography-based determinants reflect the community SES, while individual determinants are specific to the patient. After adjusting for age, sex, and tobacco use, we found that individuals from areas with lower unemployment rates presented with CT scans demonstrating smaller nodules < 6 mm (OR 0.754, *p* 0.045), while those from areas with higher unemployment rates were presented with larger nodules (OR 1.1, *p* < 0.357). Patients with noncommercial insurance were more likely to present with larger nodules > 8 mm (OR 2.181, *p* 0.016) on first presentation and less likely to have smaller nodules < 6 mm (OR 0.437, *p* 0.01). These findings highlight that even with incidental nodules, markers of poverty are associated with larger, inherently higher-risk nodules at initial presentation.

As mentioned above, there are limited studies evaluating the relationship between SES and ILNs. However, several studies have examined the relationship between poverty and lung cancer outcomes, finding that areas of higher deprivation were associated with higher lung cancer incidence and mortality [18, 24]. These subpar outcomes are likely related to high-risk smoking behaviors and less access to healthcare compared to those with higher SES [25, 26]. Additionally, individuals with lower SES may be more likely to be exposed to secondhand smoke and other environmental toxins that increase the risk of lung cancer [16]. There is growing evidence evaluating the impact of insurance type and lung cancer outcomes. A recent study evaluating ILN surveillance found that underinsured individuals were more likely to be diagnosed with cancer. Individuals with federal insurance are also less likely to complete screening [17, 27] and may not receive full coverage for LDCT.

Importantly, patients presenting with ILNs are less likely to be eligible for LCS. It is not yet known if SES disparities in LCS eligibility result in more patients with lower SES presenting with ILNs, rather than participating in LCS programs. This is a notable consideration, since patients with ILNs appear to be at a higher risk for ultimately being diagnosed with lung cancer [14]. Disparities in eligibility for LCS due to race have been described [16, 18], but a significant knowledge gap still exists with respect to this relationship between SES and ILNs. There was a period of 11 months for the Centers for Medicare and Medicaid Services (CMS) to expand LCS eligibility criteria (February 2022) to reflect the USPSTF eligibility update (March 2021) [3, 28]. Consequently, most individuals in our study period would not have benefited from the expanded criteria. CMS limits age to 77 years in determining LCS eligibility [28], although most guidelines recommend continued screening up to 80 years old [5–7]. Age is a well-established risk factor for lung cancer [2]. Our study showed that increased age was weakly associated with larger nodules > 8 mm, although with a small effect size. Taken together, these findings suggest that older individuals with federal insurance may be ineligible for LCS. These limitations may create additional barriers for LCS in individuals with low SES [16, 17].

We also evaluated less commonly studied markers of SES, such as the air quality index and cost of living index (COLI). COLI is a relative marker of living expenses compared to United States estimates, with values above 100 conferring higher than average costs of living. COLI has not been extensively studied in relation to ILN outcomes; however, prior studies in individuals with hepatocellular cancer found that those with lower COLI presented with more advanced cancers, while higher COLI was associated with improved survival [29]. We found a weak association between individuals from lower COLI areas and smaller nodules < 6 mm (OR 0.933, *p* 0.056), while higher COLI areas were weakly associated with nodules between 6–8 mm (OR 1.084, *p* 0.048), the significance of which is undetermined. Individuals with lower SES may be more likely to reside in lower COLI areas; however, New York City (NYC) on average has the highest COLI in the United States, and all individuals in this study resided in areas with a COLI > 100. Studies suggest that increased living costs may compete with other financial burdens, which may disproportionately affect individuals with lower SES, leading to less guideline concordant care [5]. Larger studies utilizing the cost of living index may help further define these relationships in individuals with lung nodules.

The air quality index is a measure of air pollution, standardized based on the Clean Air Act [30]. Metropolitan areas such as NYC are required to report air quality daily. In our study, there were no associations with nodule size and air quality index, and all individuals resided in areas with satisfactory air quality. The association of the air quality index and ILN size has not been explored, but previous studies have demonstrated significant associations with a higher air quality index and increased lung cancer incidence [31]. While no relationship was present in our study, it will be interesting to assess how SES and air quality impact ILN size in areas with more pollution.

Education level has been assessed as a risk factor for worse lung cancer outcomes. Health literacy may be lower in individuals with lower educational attainment [32], and these individuals may face more barriers to screening despite being at higher risk [33]. Our study showed that individuals from areas with educational attainment less than high school were more likely to have smaller nodules (OR 1.243, *p* < 0.008). There was no association between education and nodules > 8 mm. There are limited studies evaluating the relationship between educational status and ILNs, however prior population studies have found that educational attainment lower than high school level was associated with decreased LCS eligibility, in contrast to those with college education or higher (which compromised a significant percentage of NLST participants) [16]. Our assessment was limited by including only one measure of educational attainment, having no comparison group with those of higher attainment, and a small sample size. The association between education and ILNs should be further explored, as individuals with ILNs represent a distinct population from those in LCS programs and are less likely to be eligible for LCS.

### Race/ethnicity and nodule size

The distribution of race and ethnicity in our sample was diverse, with mostly nonwhite Hispanic individuals (56%) followed by African American (26.5%) and White (15.3%) individuals. This significantly differs from the NLST, which comprised 91% White individuals [5, 6]. Our study found that nonwhite individuals were 0.67 times less likely to have smaller nodules < 6 mm and 1.63 times more likely to have nodules > 8 mm, although these relationships were not statistically significant. It is crucial to examine the intersection of socioeconomic status (SES) and racial disparities in lung cancer outcomes, given its strong correlation. Although there is limited sociodemographic data on ILN programs in the current literature to date, studies have shown that individuals belonging to racial and ethnic minorities have the lowest SES, resulting in lower LCS utilization [16, 34]. However, there is a lack of research on how the combination of low SES and minority racial status impacts ILN surveillance, LCS eligibility, utilization, or outcomes. Other studies found that African American participants had a lower screening rate than White participants, and unscreened individuals had a lower annual household income [34]. This suggests that African American individuals with low annual household income may have an even lower screening rate. Additionally, our study showed that when adjusting for race/ethnicity, SES determinants demonstrated stronger associations with ILN size. Other studies have shown that lower SES and ethnic-minority groups have significantly lower overall lung cancer patient survival rates [2, 17]. These findings suggest that SES represents an important driver of ILN size and ultimately lung cancer risk.

### Limitations

Our study has several limitations. As with all retrospective studies, our data reveal associations but does not provide evidence for causation. Several SES determinants, such as income and poverty level, did not have significant relationships with lung nodule size. This may be due to the overall high rate of deprivation in the population served by the safety-net hospital, making it more difficult to find significant relationships, since most areas may be similarly deprived.

As a community hospital located in an area facing a shortage of healthcare professionals during the COVID-19 pandemic, our center did not have an established formal surveillance program for LCS or ILNs during the study period. We included individuals with ILNs who were referred to the pulmonary service for any indication. However, primary care providers in the community may manage ILNs without referring to the pulmonary service. Since our study only included patients referred to the pulmonary services, patients with ILNs managed by PCPs were excluded, which reduced the power of the study. It is conceivable that variability in the medical and socioeconomic history of these excluded patients, compared to those referred to the pulmonary service, could have influenced the findings.

Conversely, our study included many patients with small, lower risk ILNs. This distinguishes our study from others that include patients from formal LCS or ILN programs, which generally include nodules at least 6 mm in size. Nonetheless, our sample represents a socially diverse at-risk group that is typically excluded from research studies.

A complete case analysis approach was utilized, which resulted in a significant number of patients with nodules being excluded due to missing nodule size or incomplete description. It is unlikely this nodule data were missing at random. It is possible that radiologists were less likely to comment on size for small, less clinically significant nodules. Since nodule size was an outcome of interest, and the nodule data were unlikely to be missing at random, the complete case analysis is biased.

Additionally, geographical markers of socioeconomic status were derived from census data according to zip code. These markers do not reflect the individual socioeconomic status of patients but rather reflect the deprivation faced by their neighborhoods. The individual patient may have markers of higher or lower SES compared to their neighborhood.

## Conclusion

In conclusion, high-risk individuals presenting with ILNs represent a distinct but complementary at-risk population for lung cancer to those being screened, and combined nodule surveillance and LCS programs lead to improved guideline concordant care. In our unique cohort of patients in a socioeconomically deprived area without a formal LCS program, we found that individuals with lower SES defined by geographical and individual determinants present with larger incidental nodules. This finding confers higher risk to such patients undergoing lung cancer evaluation and underscores the inequity that exists for patients with socioeconomic disadvantage. Individuals with markers of higher SES, in contrast, were more likely to have smaller nodules at presentation. While the association of SES with LCS has been heavily explored, these data suggest that SES also impacts evaluation of incidental lung nodules, filling a key knowledge gap in the present literature. The cost of living index and air quality were also investigated as less commonly used determinants of SES. Further studies with these indices may help delineate their relationships with ILN size.

## Data Availability

The datasets used and/or analyzed during the current study are available from the corresponding author on reasonable request.

## Abbreviations

ADI: Area Deprivation Index
COPD: Chronic obstructive pulmonary disease
HIV: Human immunodeficiency virus
COVID-19: Coronavirus disease 2019
SES: Socioeconomic status
IRB: Institutional review board
OR: Odds ratio
NLST: National lung cancer screening trial
NELSON: Nederlands–Leuvens Longkanker Screenings Onderzoek
CT: Computed Tomography
LDCT: Low-dose computed tomography
ADI: Area Deprivation Index
US: United States
USPSTF: United States Preventive Services Task Force
ILN: Incidental lung nodule
LCS: Lung cancer screening
CMS: Centers for Medicare and Medicaid Services
NYC: New York City
NYCHHC: New York City Health and Hospitals
STROBE: Strengthening the reporting of observational studies in epidemiology
